# A platelet-mimicking theranostic platform for cancer interstitial brachytherapy

**DOI:** 10.7150/thno.61259

**Published:** 2021-06-04

**Authors:** Meng Lyu, Mingzhu Chen, Lujie Liu, Daoming Zhu, Xianjia Wu, Yang Li, Lang Rao, Zhirong Bao

**Affiliations:** 1 Department of Radiation and Medical Oncology, Hubei Key Laboratory of Tumor Biological Behaviors, Hubei Cancer Clinical Study Center, Zhongnan Hospital of Wuhan University, Wuhan 430071, China.; 2 Institute of Biomedical Health Technology and Engineering, Shenzhen Bay Laboratory, Shenzhen 518132, China.; 3 Department of Gastrointestinal Surgery, Second Clinical Medical College of Jinan University, Shenzhen People's Hospital, Shenzhen 518020, China.

**Keywords:** biomimetic, radiosensitization, brachytherapy, colon cancer, hypoxia

## Abstract

**Rational:** Interstitial brachytherapy (BT) is a promising radiation therapy for cancer; however, the efficacy of BT is limited by tumor radioresistance. Recent advances in materials science and nanotechnology have offered many new opportunities for BT.

**Methods:** In this work, we developed a biomimetic nanotheranostic platform for enhanced BT. Core-shell Au@AuPd nanospheres (CANS) were synthesized and then encapsulated in platelet (PLT)-derived plasma membranes.

**Results:** The resulting PLT/CANS nanoparticles efficiently evaded immune clearance and specifically accumulated in tumor tissues due to the targeting capabilities of the PLT membrane coating. Under endoscopic guidance, a BT needle was manipulated to deliver appropriate radiation doses to orthotopic colon tumors while sparing surrounding organs. Accumulated PLT/CANS enhanced the irradiation dose deposition in tumor tissue while alleviating tumor hypoxia by catalyzing endogenous H_2_O_2_ to produce O_2_. After treatment with PLT/CANS and BT, 100% of mice survived for 30 days.

**Conclusions:** Our work presents a safe, robust, and efficient strategy for enhancing BT outcomes when adapted to treatment of intracavitary and unresectable tumors.

## Introduction

Colon cancer, with an overall survival rate as low as 14% when diagnosed at a high-risk stage, is one of the most common human malignancies 1. In individuals with recurrent or metastatic colon cancer, tumors typically invade adjacent or distant organs, thus surgical treatment is often unable to cure the underlying disease 2. Older patients are additionally less able to tolerate such surgical procedures, limiting their potential for positive outcomes 3, 4. For patients who are not eligible for curative tumor resection, radiotherapy (RT) has gradually become a salvage treatment strategy due to its relative non-invasiveness and highly efficient therapeutic effect 5.

In the clinic, there are generally two types of RT: external beam radiotherapy (EBRT) and brachytherapy (BT). In EBRT, high-energy radioactive beams are delivered to the tumor site from outside the body using a linear accelerator or ^60^Co 6, during which absorption of radiation energy by normal soft tissues in the path of the radiation beam inevitably results in severe side effects 7, 8. Additionally, accurate tumor localization is limited by respiratory motion 9, organ movement 10, daily variability 11, and setup error. In comparison, BT systems directly transfer therapeutic radioisotopes such as ^131^I 12-14 or ^192^Ir 15 into the tumor regions using a minimally invasive afterload instrument. Thus, BT presents dosimetric advantages by realizing targeted delivery of radioisotopes, providing high-dose-rate irradiation dose deposition specifically to tumors while limiting the dosage in surrounding healthy tissues. For this reason, BT optimizes the therapeutic effects in tumors while avoiding systemic radiotoxicity to normal organs and alleviating the suffering of patients 18. BT is recommended for the treatment of prostate cancer 19, 20, cervical cancer 21, and other types of cancers 22, 23.

Despite its use in cancer treatment, the efficacy of BT is still limited by the radioresistant characteristics of many solid tumors 24-27. As tumors are composed of rapidly proliferating cells associated with an abnormal blood supply, tumor growth may outpace the corresponding oxygen and nutritional supply system, resulting in significant intratumoral hypoxia (pO_2_ < 1.3%) 16. Oxygen, however, is essential to enhancing radiation-induced DNA damage; therefore, hypoxia-associated radioresistance occurs in many types of solid malignant tumors 17-19. Various approaches have been devised to either alleviate or leverage such intratumoral hypoxia 20-22. Magnetic hyperthermia and photothermal therapy approaches increase the intra-tumoral temperature and blood flow, thus improving oxygen delivery to the tumor site 23-25. Tirapazamine, in contrast, is a prodrug that is activated under hypoxic conditions and can be administered as a complementary approach together with radiotherapy to treat hypoxic tumors 26, 27. Other researchers have explored direct intratumoral oxygen delivery strategies using hemoglobin 28, perfluorocarbon-based nanoplatforms 24, or enzyme-like nanoparticles capable of mediating *in situ* oxygenation by degrading endogenous H_2_O_2_ and, thereby, overcoming hypoxia 29, 30.

**Scheme 1:**
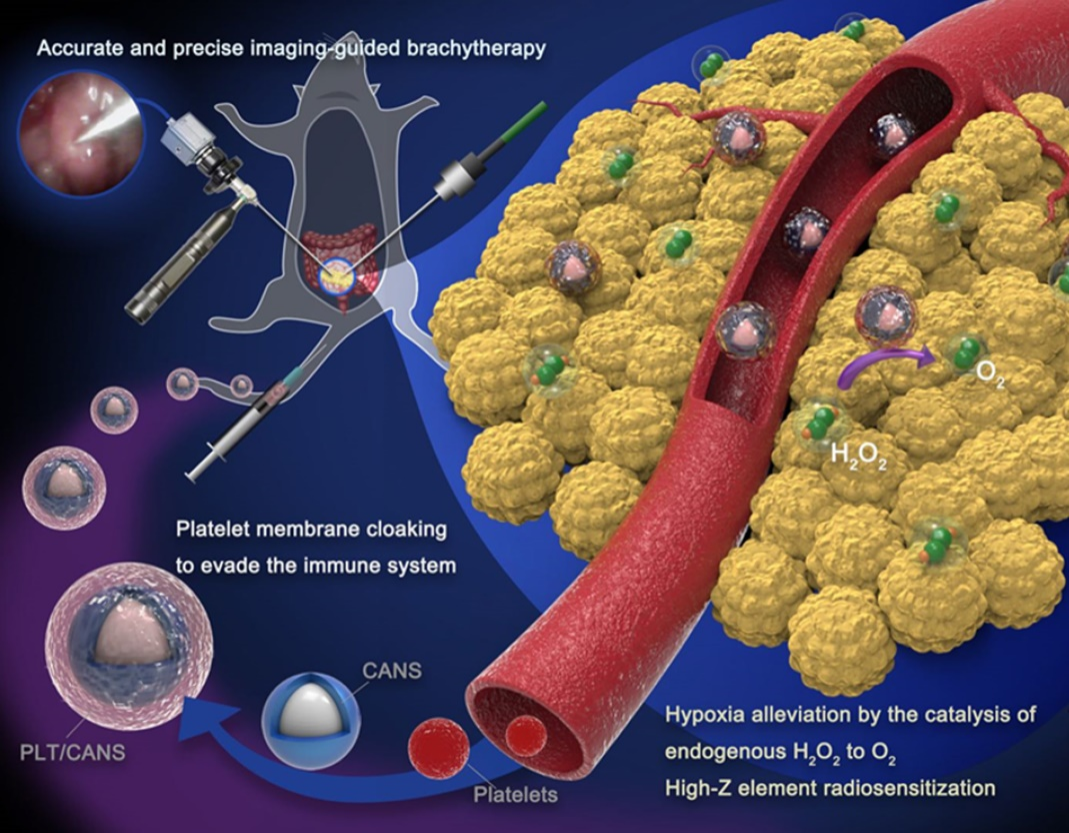
PLT/CANS preparation and an overview of endoscope-guided interstitial BT. The PLT membrane-cloaked nanoparticles target tumor regions, alleviating hypoxia by catalyzing intratumor H_2_O_2_ to O_2_ and depositing energy via their high-Z elements.

Effective tumor radiosensitization depends on reliable nanoparticle targeting and enrichment in the tumor 31-33. In an effort to enhance the efficacy of interstitial BT in a murine model of colon cancer, we developed platelet (PLT) membrane-coated core-shell Au@AuPd nanospheres (PLT/CANS) and employed them as radiosensitization agents. We hypothesized that the PLT-mimicking properties of these nanoparticles would enable them to avoid macrophage-mediated clearance and selectively target abnormal intratumoral vascular structures 34-41, resulting in nanoplatform enrichment within the tumor (Scheme 1). In contrast, red blood cell (RBC) membrane-coated nanoplatforms can effectively evade immune cell-mediated clearance 42, 43, but lack the ability to specifically target tumor tissues. The high-Z elements gold and palladium are ideal radiosensitizers that enhance BT by depositing irradiation energy in the tumor. Palladium-based PLT/CANS can also catalyze H_2_O_2_ degradation to increase tumor oxygenation, thereby alleviating hypoxia and overcoming tumor radioresistance. Therefore, the biomimetic nanotheranostic platform developed in this study for enhanced interstitial brachytherapy could lead to selective cancer cell killing while minimizing the risk to surrounding organs and tissues. This novel strategy has great potential for the treatment of colon cancer as well as other abdominal and unresectable cancer types.

## Methods

### Materials

60 nm gold nanospheres were purchased from BBI Solutions (England). Sodium tetrachloropalladate (II) (Na_2_PdCl_4_), ascorbic acid (≥99%), and chloroauric acid hydrate (AuCl_4_·4H_2_O, 47.8%) were purchased from Aladdin Reagent Co., Ltd. (Shanghai, China). Phosphate-buffered saline (PBS), RPMI-1640, fetal bovine serum (FBS), dimethyl sulfoxide, and trypsin-EDTA (0.25%) were purchased from Gibco (USA). Deionized water was prepared using a Millipore System (18 MΩ·cm). Cell counting kit 8 (CCK-8), crystal violet staining solution, 4% paraformaldehyde, and fluorescein isothiocyanate were obtained from Thermo Fisher (USA).

### CANS, RBC/CANS and PLT/CANS preparation

CANS were prepared by adding 2 mL of a Na_2_PdCl_4_ (1 mM) solution and 0.1 mL of AuCl_4_·4H_2_O (200 mM) to 1 mL of gold nanospheres (50 mg/mL). The solution was stirred until clear, after which 1 mL of ascorbic acid (1 M) was added, and the reaction was allowed to rest undisturbed for 24 h at 25 °C. The solution was then centrifuged for 10 min at 11,000 rcf, and the particles were further purified by several washes with deionized water. The resultant product was then dried for 8 h in a vacuum oven.

RBC membranes were obtained via a previously reported low-osmosis method 44. RBC membrane vesicles derived from 10 μL of murine blood were first mixed with 50 μg of CANS in 1 mL of PBS, after which the mixture was extruded through a 200 nm Nuclepore polyester membrane in a mini-extruder. The resultant particles were then spun for 10 min at 1,000 × *g* to remove remaining RBC-derived vesicles, and the resultant RBC/CANS were collected and stored at 4 °C in PBS for subsequent experiments.

A similar approach was used to synthesize PLT/CANS. Briefly, platelets derived from whole blood were collected via gradient centrifugation by first spinning 10 mL of whole murine blood at 100 × *g* for 20 min. The supernatant was then spun at 800 × *g* for 20 min. The platelets were then repeatedly washed with PBS. All subsequent steps were identical to those used to synthesize RBC/CANS.

### Cell culture and viability measurements

RAW 264.7 and CT 26 cells were cultured in DMEM containing 10% FBS at 37 °C in a 5% CO_2_ incubator. Cytotoxicity assays were conducted using RAW 264.7 cells in the logarithmic phase of growth that were seeded in 96-well plates at 5 × 10^3^ cells per well and then incubated for 24 h, after which a CCK-8 kit was used to assess cell viability. All analyses were conducted in triplicate.

### Orthotopic colon cancer model

All animal experiments were performed according to the protocols of the Institutional Animal Care and Use Committee (IACUC) of the Animal Experiment Center of Wuhan University (Wuhan, China). BALB/c mice were anesthetized via intraperitoneal injection of 1% pentobarbital then fixed in a supine position. Hair was removed from the surgical site, and the skin was disinfected with iodophor and 95% ethanol. A longitudinal incision was then made at the lower margin of the abdominal line, and the skin and peritoneum were incised to expose the bladder and small intestine, which were removed using forceps to expose the large intestine deep within the abdomen. A 25 μL CT 26 cell suspension (1 × 10^8^ cells/mL) was then injected into the colon submembrane space while the colon was held with ophthalmic tweezers. The colon was held momentarily after injection, after which the needle was slowly removed and the injection site was covered with sterile gauze for 10 s to ensure that the liquid remained *in situ.* The abdominal cavity was then closed.

### Antitumor efficacy

Orthotopic tumor-bearing mice were randomly assigned to six different treatment groups 7 days after tumor model establishment (*n* = 5/group): 1. PBS (150 μL), 2. BT (6 Gy), 3. RBC/CANS (2.5 mg/kg), 4. PLT/CANS (2.5 mg/kg), 5. RBC/CANS (2.5 mg/kg) + BT (6 Gy), and 6. PLT/CANS (2.5 mg/kg) + BT (6 Gy). All nanomaterials were intravenously injected into treated mice. Mice in groups 5 and 6 underwent irradiation at 6 h following nanoparticle injection. Before irradiation, mice were anesthetized using 4% isoflurane and an endoscope was used to visualize the tumor. The endoscopic probe was inserted from the right probe, while the BT implant probe was inserted from the left side. The irradiation was conducted via an implanted probe connected to an afterload system, which utilizes ^192^Ir as the irradiation source. Mice were monitored for up to 56 days, at which time all surviving mice were euthanized. In a second experiment, mice were assigned to the same treatment groups but were euthanized at 14 days post-treatment, at which time colon tissues were surgically excised and examined.

### Statistical analysis

Data were compared via one-tailed Student's t-tests, with *P* < 0.05 as the significance threshold.

## Results and Discussion

Our approach to PLT/CANS synthesis is shown in Scheme 1. Core-shell Au@AuPd nanospheres (CANS) were encapsulated in PLT-derived membranes. Due to the properties of PLT membrane, intravenously administered PLT/CANS were expected to target damaged vascular tissues within tumors and accumulate. Interstitial BT could then be conducted under endoscopic guidance. Overall, coating CANS with PLT-derived membrane material was expected to improve the accumulation of CANS within tumors while shielding them from immune cell-mediated clearance and, thereby, prolonging their circulation.

**Figure 1:**
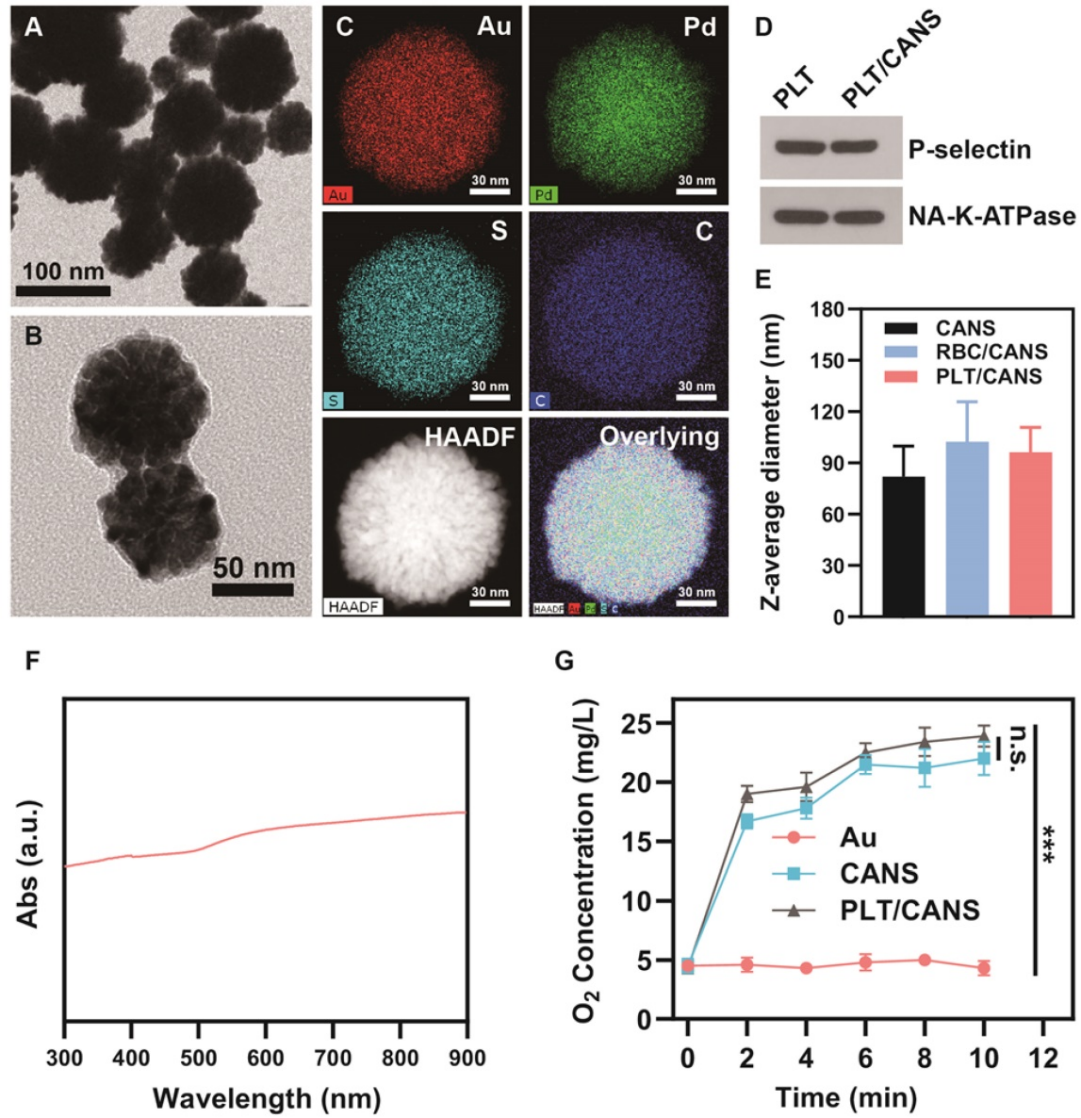
Characterization of PLT/CANS. TEM images of A) CANS and B) PLT/CANS. C) Element mapping of PLT/CANS. D) Representative Western blots of protein in PLT and PLT/CANS. E) Hydrodynamic diameters of CANS, RBC/CANS, and PLT/CANS by DLS. F) UV-vis absorption spectrum of PLT/CANS (200 µg/mL). G) Oxygen concentration over time in equivalent solutions of Au, CANS, and PLT/CANS. *** *P* < 0.01.

Transmission electron microscopy (TEM) analyses revealed that the prepared CANS exhibited a core-shell spherical structure with a diameter of 80 ± 18 nm (Figure 1A). Moreover, the diameter of the Au core was ~60 nm, as shown in Figure 1S, indicating that the thickness of the AuPd alloy shell was ~20 nm. X-ray photoelectron spectroscopy (XPS) analyses with high resolution of a single element confirmed the valences of the gold and palladium used in this study (Figure S2A and S2B). Figure S2C shows the XPS survey results, indicating the existence of gold and palladium elements. From these XPS results, it was concluded that gold was in a simple Au form, while palladium was in a complex form consisting of Pd, PdO, and PdOx. Successfully prepared PLT/CANS were encapsulated in an ~8.9 nm-thick lipid bilayer, as shown in Figure 1B. Elemental mapping results were consistent with a successful PLT coating, revealing the presence of carbon and sulfur as the primary membrane components (Figure 1C). The coating was also associated with a shift in zeta potential from -19.5 mV to -25.6 mV, the latter being close to the zeta potential of PLT membrane (Figure S2D). Western blotting confirmed the presence of PLT-derived proteins in PLT/CANS, including P-selectin and NA^+^/K^+^-ATPase (Figure 1D). Meanwhile, sodium dodecyl sulphate polyacrylamide gel electrophoresis results further confirmed the successful coating of PLT membrane on CANS (Figure S2E). As a control particle, we additionally prepared CANS particles encapsulated in RBC membranes (RBC/CANS) (Figure S3). The average diameters of RBC/CANS and PLT/CANS were 20.5 nm and 14.3 nm larger than uncoated CANS, respectively, as measured by dynamic light scattering (DLS) (Figure 1E). The UV-vis absorption spectrum of PLT/CANS exhibited strong absorption across a wide spectral range (Figure 1F).

Palladium-based nanoparticles have previously been reported to promote O_2_ production by catalyzing endogenous H_2_O_2_ within tumors 45. To explore the catalytic activity of PLT/CANS, we measured O_2_ generation in H_2_O_2_ solution after adding 60 nm gold spheres (Au), CANS, or PLT/CANS. We observed that oxygen levels remained almost constant after the addition of Au. In comparison, the O_2_ concentration continuously increased to 22.0 mg/L and 23.9 mg/L after addition of CANS and PLT/CANS, respectively (Figure 1G), confirming the efficient catalytic activity of these particles and indicating that the PLT membrane coating had no adverse impact on such catalytic activity. Therefore, PLT/CANS holds potential to alleviate hypoxia in tumor sites.

**Figure 2:**
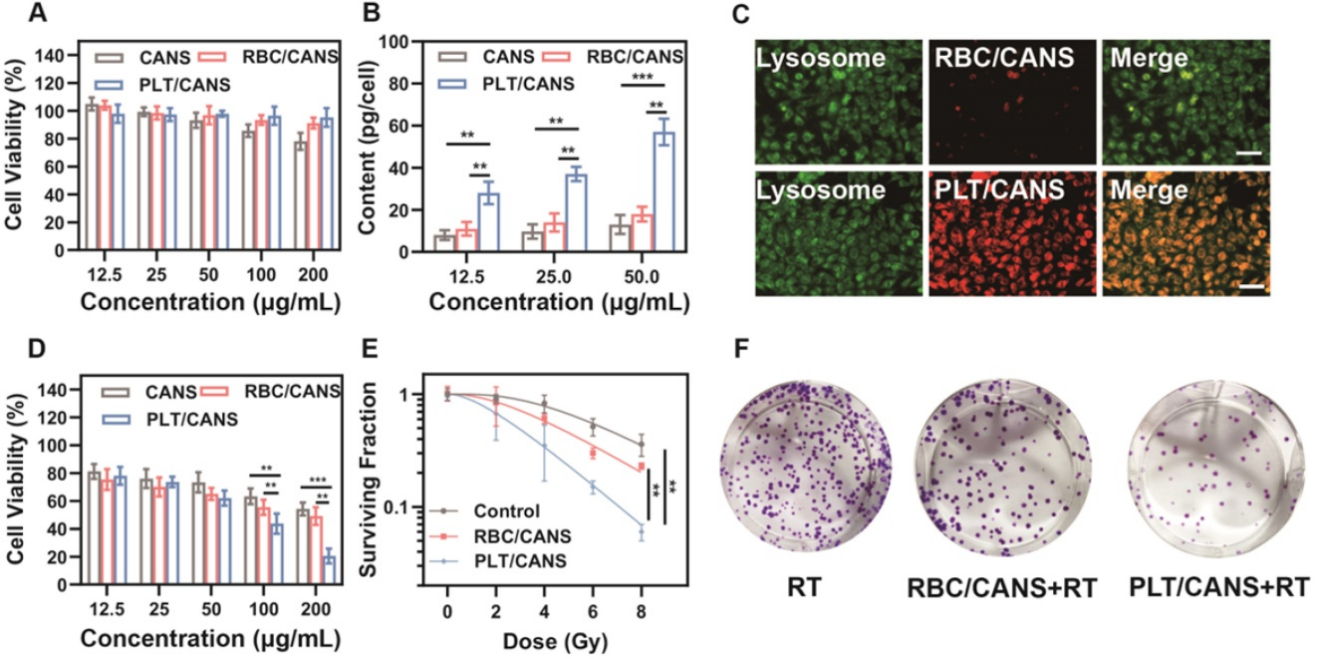
*In vitro* assessment of CANS, RBC/CANS, and PLT/CANS. A) Cell viability of RAW 264.7 cells treated with the indicated nanoparticle doses. B) Au levels in CT 26 cells treated with the indicated nanoparticle doses. C) Fluorescence images of CT 26 cells treated with Cy5-labeled RBC/CANS or PLT/CANS (50 μg/mL). Scale bars: 50 μm. D) Cell viability of CT 26 cells incubated with the indicated nanoparticle doses and then irradiated with 6 Gy. E) Results of a colony formation assay assessing treatment outcomes at a range of radiation doses. F) Images of a colony formation assay at a radiation dose of 6 Gy. ** *P* < 0.01; *** *P* < 0.001.

The cytotoxicity of the prepared nanoparticles was then evaluated using CCK-8 assays. RAW 264.7 cells were incubated with various concentrations of CANS, RBC/CANS, or PLT/CANS for 24 h. The CCK-8 results shown in Figure 2A reveal that cells treated with RBC/CANS or PLT/CANS had no observable cytotoxic response even at high nanoparticle concentrations up to 200 μg/mL, indicating that both the PLT and RBC membrane coatings markedly improved the biocompatibility of CANS and reduced the possible cytotoxicity of these nanoparticles. Next, we analyzed the uptake of the nanodrugs in CT 26 cells using inductively coupled plasma atomic emission spectroscopy. After 4 h of incubation, both RBC/CANS and naked CANS were taken up by cells to significantly lower levels than PLT/CANS (Figure 2B and 2C). The Au content in the PLT/CANS-treated cells was maintained at a high level from 6 to 12 h post treatment (Figure S4A). In addition, the immune escape ability of PLT/CANS was verified by macrophage uptake analysis. By detecting the Au content in RAW 264.7 cells, we found that PLT/CANS and RBC/CANS had low uptake while naked CANS exhibited a high degree of uptake (Figure S4B). Interestingly, the cellular uptake of PLT/CANS was obviously lower than that of RBC/CANS, indicating the superior immune escape ability of PLT/CANS. Thus, we confirmed that the prepared PLT/CANS successfully inherited the excellent biocompatibility and immune evasion functionality of PLT membrane.

To investigate the radiation sensitivity of these nanoparticles *in vitro*, we pretreated CT 26 tumor cells with a range of nanoparticle concentrations and then delivered radiation at a dose of 6 Gy. As shown in Figure 2D, cells pretreated with PLT/CANS exhibited greater growth inhibition compared with the RBC/CANS and CANS groups, probably because the high cellular uptake of PLT/CANS led to enhanced radiosensitivity. A colony formation assay was then performed to further explore the cellular response. The results shown in Figure 2E reveal a significant (*P* < 0.01) reduction in viability for those cells treated with RBC/CANS or PLT/CANS compared to the control group that only received RT. Representative images of the above experiment are presented in Figure 2F. When the resultant data were fit to a “multitarget-single-hit” model, the sensitization enhancement ratio (SER) values for RBC/CANS and PLT/CANS were calculated to be 1.07 and 1.48, respectively. Western blotting was conducted to confirm the occurrence of DNA double-strand breaks and assess the DNA repair ability of the three groups (Figure S5). We found that the expression of γ-H2AX was increased while the expression of the DNA double-strand break repair protein RAD51 was decreased in cells treated with PLT/CANS + RT compared to RBC/CANS + RT or RT alone, indicating severe DNA double-strand breaks and limited repair ability in the PLT/CANS group. Thus, our results strongly suggest that PLT/CANS can markedly enhance tumor cell sensitivity to irradiation *in vitro*.

**Figure 3:**
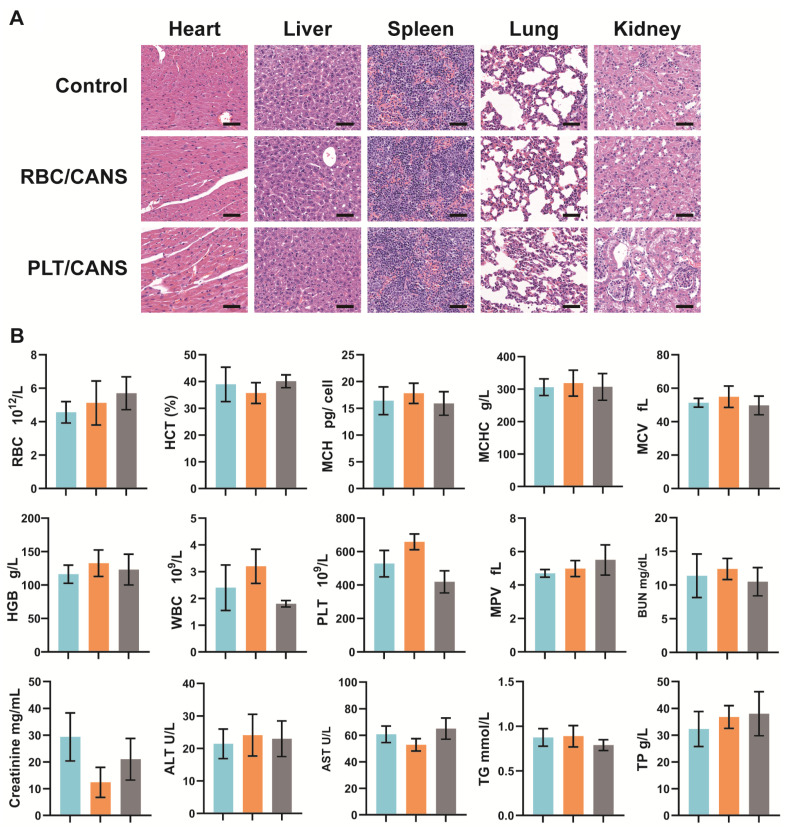
*In vivo* biocompatibility of CANS, RBC/CANS, and PLT/CANS. A) H&E staining of primary organs. Scale bars: 50 µm. B) Hematological analyses of mice treated with saline (blue), RBC/CANS (orange), or PLT/CANS (gray).

Before investigating the ability of the nanoparticles to enhance the *in vivo* anticancer efficacy of BT, their long-term biosafety was assessed. Healthy BALB/c mice were intravenously injected with CANS, RBC/CANS, or PLT/CANS and then sacrificed 60 days after treatment. Major organs including heart, liver, spleen, lungs, and kidneys were collected for H&E staining analysis. Mice untreated with nanoparticles were used as controls. No pathological abnormalities were observed in any of the treated groups (Figure 3A), indicating their possible nontoxicity *in vivo*. In addition to histological examination, we carried out serum biochemistry and hematology studies to look for any potential toxicity of our nanoparticles in mice. Consistent with the above results, whole blood analysis, including RBC counts, hematocrit (HCT), mean corpuscular hemoglobin (MCH), mean corpuscular hemoglobin concentration (MCHC), mean cell volume (MCV), hemoglobin (HGB) counts, white blood cell (WBC) counts, PLT counts, and mean PLT volume (MPV), were within normal ranges for all groups (Figure 3B). Meanwhile, liver function markers, including alanine aminotransferase (ALT) and aspartate aminotransferase (AST), and kidney function markers, including creatinine, blood urea nitrogen (BUN), total protein (TP), and triglycerides (TG), were normal, indicating that the nanoparticles caused no significant renal or hepatic toxicities. In addition, hemolysis tests confirmed that CANS, RBC/CANS, and PLT/CANS possess good biocompatibility (Figure S6). These preliminary but comprehensive assessments suggest that PLT/CANS may have acceptable biocompatibility *in vivo*.

**Figure 4:**
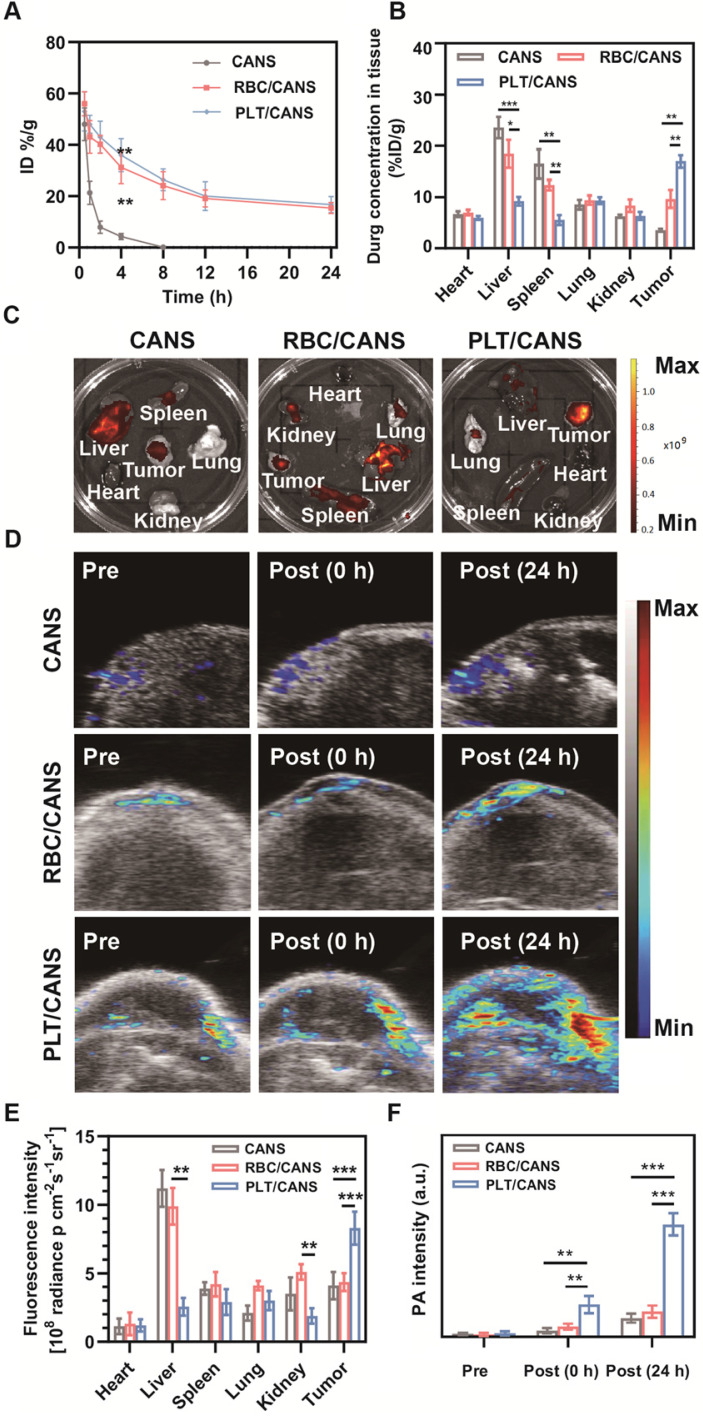
Tumor-specific accumulation of PLT/CANS. A) Nanoparticle blood clearance in healthy mice. B) Nanoparticle biodistribution at 24 h postinjection in an orthotopic colon cancer model. C) Fluorescence images of major organs and tumors at 24 h postinjection. D) PA images of tumors before and after nanoparticle administration. E) Semiquantitative analysis of the fluorescence imaging signal in major organs and tumors at 24 h postinjection. F) Semiquantitative analysis of the PA signal in the tumor before and after nanoparticle administration. * *P* < 0.1; ** *P* < 0.01; *** *P* < 0.001.

We next established an orthotopic colon cancer model to examine the specific accumulation of PLT/CANS within tumors. Firstly, we monitored the circulation profiles of the nanoparticles in healthy mice following intravenous injection. Both RBC/CANS and PLT/CANS exhibited similarly prolonged circulation relative to uncoated CANS, which were rapidly cleared and thus unsuitable for therapeutic utilization (Figure 4A). We next monitored the tumor tissues of mice from 0 to 48 h after nanoparticle injection, and found that the mice administered PLT/CANS exhibited the highest tumor accumulation among the three groups (Figure S7). The prolonged circulation of the membrane-coated nanoparticles may be due to reduced CD47-dependent cellular internalization 34, 42, 46. This long circulation would further enable the membrane-coated nanoparticles to take advantage of the enhanced permeability and retention effect in tumors to achieve passive tumor targeting. To quantitatively confirm this tumor-targeting ability, accumulation of nanoparticles in the tumor and major organs was assessed at 24 h post-administration by measuring the tissue Au content via inductively coupled plasma mass spectrometry. As shown in Figure 4B, the biodistribution profiles revealed that PLT/CANS accumulated more effectively in tumors than RBC/CANS and CANS, which primarily localized to the liver. These quantitative results are consistent with results from *ex vivo* fluorescence imaging (Figure 4C). The fluorescence intensity of PLT/CANS in the tumor was ~1.90-fold higher than that of RBC/CANS (Figure 4E), suggesting that PLT/CANS accumulated in the tumor more effectively. To further assess the time dependence of nanoparticle tumor accumulation, mice bearing subcutaneous CT 26 tumors were imaged by photoacoustic (PA) imaging at the specific wavelength absorbed by CANS. As shown in Figure 4D and 4F, there was a slight increase in the intratumoral PA signal at 24 h in mice injected with CANS or RBC/CANS, whereas this signal was markedly enhanced in mice treated with PLT/CANS owing to its excellent tumor-targeting efficiency. These PA results are consistent with the results from our *ex vivo* fluorescence imaging and biodistribution studies. Hence, we concluded that the tumor accumulation of PLT/CANS remained at a satisfactory level even at 24 h postinjection.

**Figure 5:**
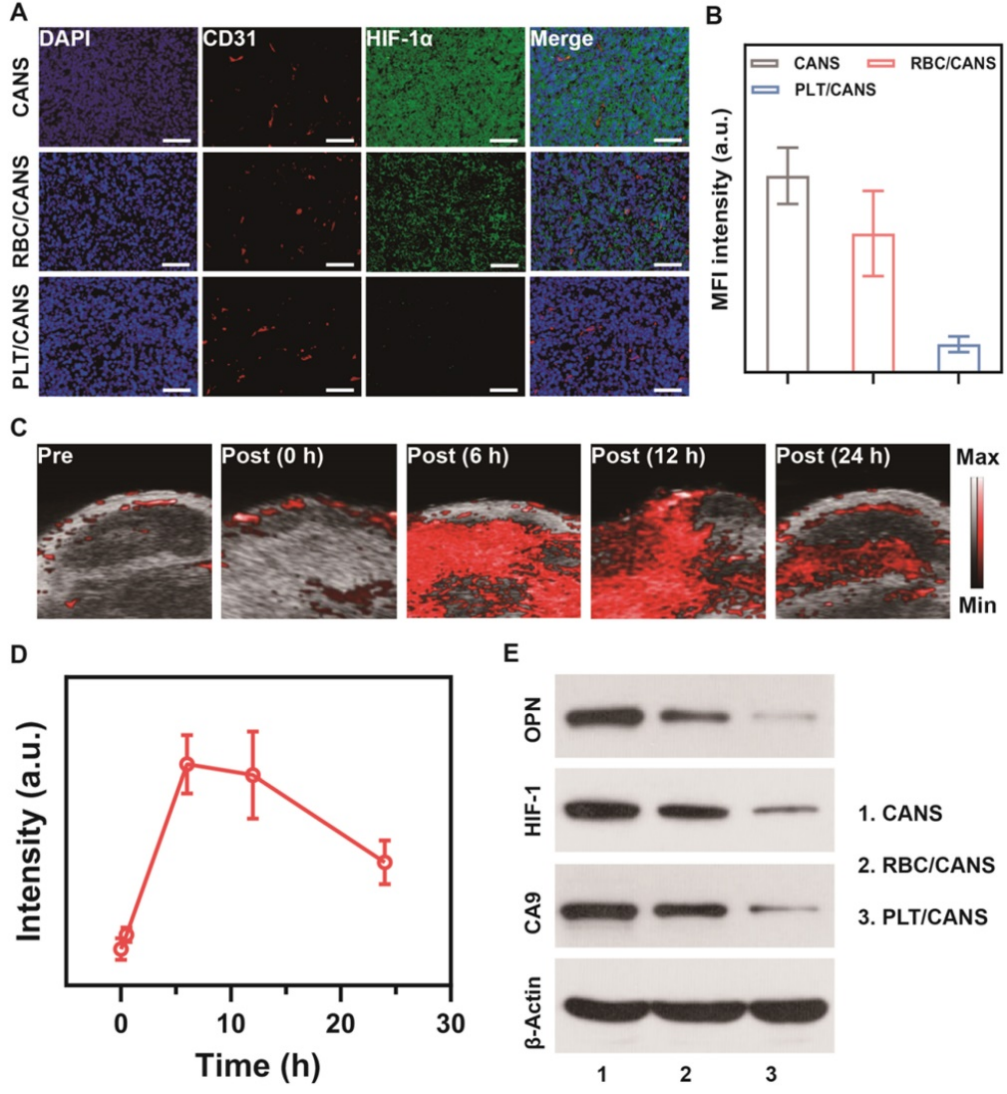
Nanoparticle-mediated alleviation of intratumoral hypoxia. A) HIF-1α staining of tumor sections collected 24 h after nanoparticle administration. Scale bars: 50 μm. B) Quantification of median fluorescence intensity of (A). C) Photoacoustic imaging of tumor oxygen levels before and after administration of PLT/CANS. D) Semi-quantitative analysis of (C) using ImageJ. E) Western blots of OPN, HIF-1, and CA9 in tumor tissue from nanoparticle-treated mice.

Inspired by the effective tumor accumulation of PLT/CANS shown above, we next investigated its ability to relieve tumor hypoxia *in vivo*. Orthotopic tumor-bearing mice were intravenously injected with RBC/CANS or PLT/CANS and sacrificed at 24 h, and then tumor hypoxia was evaluated by HIF-1α immunofluorescence staining. Intratumoral hypoxia identified by positive HIF-1α staining (green) was evident in the control group, whereas this signal was significantly reduced in mice administered RBC/CANS and further decreased in mice administered PLT/CANS (Figure 5A and 5B). This result indicates that tumor hypoxia was efficiently relieved by PLT/CANS via catalyzation of endogenous H_2_O_2_ within the tumor microenvironment to produce O_2_. The fluorescence intensity of the HIF-1α-positive areas of the tumor samples from the RBC/CANS group was 4.93-fold higher than that of the PLT/CANS group, further supporting the superior ability of PLT/CANS to alleviate intratumoral hypoxia *in vivo*. Moreover, the oxygenation-promoting ability of PLT/CANS was further investigated using PA imaging. The typical time dependence of tumor oxygenation following PLT/CAN administration is shown in Figure 5C and semi-quantitatively analyzed in Figure 5D. No obvious change in PA signal was observed at 30 min post-injection, but the signal intensity increased dramatically at 6 h. From 6 to 12 h post injection, the oxygen status in the tumor stayed at a relatively high level. At 24 h after injection, the oxygen content was still increased relative to baseline. This alleviation of tumor hypoxia from 6 to 12 h postinjection was further confirmed by HIF-1α staining (Figure S8). Moreover, western blotting was conducted to assess the tumor tissue levels of the hypoxia biomarkers osteopontin (OPN), hypoxia-inducible factor 1 (HIF-1), and carbonic anhydrase 9 (CA9). As demonstrated in Figure 5E, the three hypoxia indicators were downregulated in mice administrated RBC/CANS or PLT/CANS. Weaker signals were observed in the PLT/CANS group, indicating that tumor hypoxia was more efficiently relieved by PLT/CANS than RBC/CANS. From these tissue oxygenation results and the above tumor accumulation results, we concluded that RT should be conducted at 6 to 12 h postinjection to gain the best treatment effect.

**Figure 6:**
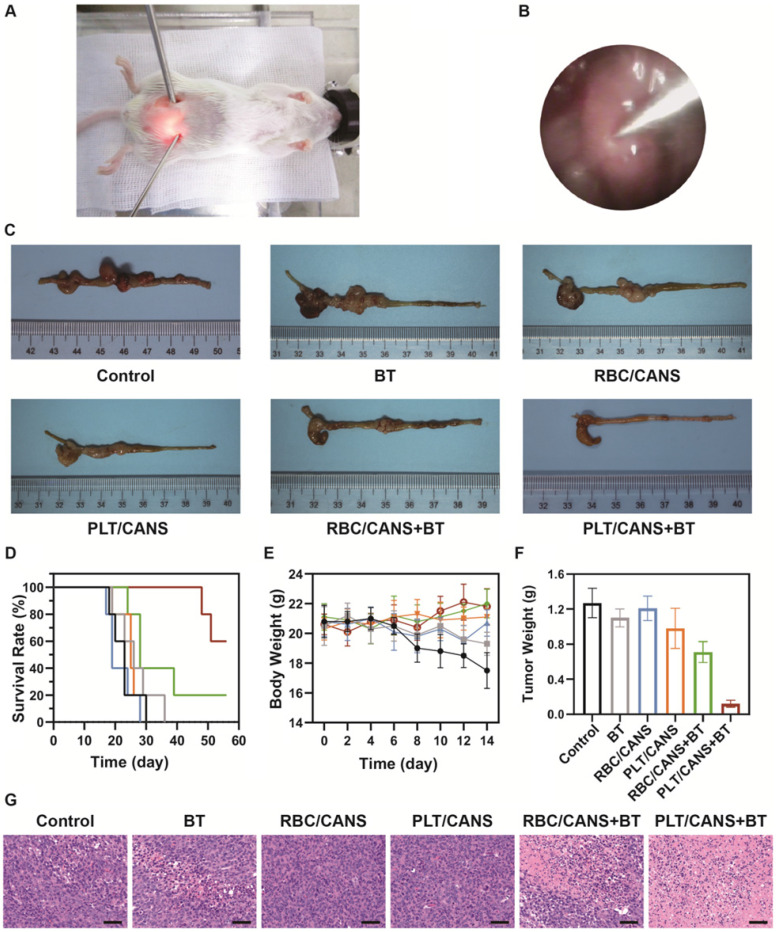
Antitumor efficacy of PLT/CANS in mice bearing orthotopic colon tumors. A) Representative image of a mouse undergoing BT under endoscopic guidance. B) Real-time image of a colon tumor visualized using an endoscope. C) Excised colons from treated mice. D) Survival rates, E) body weights, and F) tumor masses of mice in the indicated treatment groups. G) H&E-stained tumor sections. Scale bars: 50 μm. Black line: control; gray line: BT; blue line: RBC/CANS; orange line: PLT/CANS; green line: RBC/CANS + BT; red line: PLT/CANS + BT.

Lastly, we assessed the ability of PLT/CANS to enhance the *in vivo* anticancer efficacy of BT. Mice bearing orthotopic colon tumors were intravenously administered PBS, RBC/CANS, or PLT/CANS and then treated with BT at 6 h postinjection. A representative image of a mouse undergoing BT with endoscopic guidance is shown in Figure 6A. Real-time endoscopic images were used to visualize the tumor so as to enable manipulation of the implant probe to the tumor site while avoiding surrounding normal tissues (Figure 6B). 14 days after treatment, excised colon tissues from the mice were imaged (Figure 6C). The groups treated with saline, BT, RBC/CANS, PLT/CANS, or RBC/CANS + BT exhibited clear evidence of tumor growth, while the PLT/CANS + BT group showed more significant tumor growth inhibition. The survival of mice in these treatment groups was also monitored. 100% of mice in the PLT/CANS + BT group survived for 30 days after treatment compared to 40%, 20%, 0%, 0%, and 0% of mice in the RBC/CANS + BT, BT, PLT/CANS, RBC/CANS, and saline treatment groups, respectively (Figure 6D). Murine body weight was recorded every two days to assess overall treatment-associated toxicity (Figure 6E). Body weight decreases were observed in the saline, BT, PLT/CANS, and RBC/CANS treatment groups, which may be attributed to the rapid growth of tumors in these animals. The PLT/CANS + BT group exhibited the most apparent therapeutic effect, as evidenced by colon imaging results (Figure 6C) and by the fact that the tumor mass was lower in these mice than in mice from any other treatment group (Figure 6F). Histological analysis of tumor tissues further indicated that the PLT/CANS + BT treatment most effectively induced cellular apoptosis (Figure 6G).

## Conclusions

In summary, we developed a PLT-mimicking nanotheranostic platform as a novel strategy for enhancing anticancer interstitial BT. Specifically, owing to the catalytic activity of palladium toward endogenous H_2_O_2_, Au@AuPd nanospheres were employed for production of O_2_ and, thereby, relief of tumor hypoxia. By taking advantage of the high-Z elements effect arising from gold and palladium, CANS served as radiosensitizers to enhance dose deposition within tumors. With a PLT membrane coating, PLT/CANS exhibited inherent PLT-like properties including prolonged circulation and tumor-specific targeting, which further enhanced RT outcomes. This PLT-mimicking nanoplatform achieved satisfactory therapeutic efficacy *in vivo* upon combination with imaging-guided BT. This novel radiosensitization approach to enhancing BT efficacy is, therefore, a promising solution to the treatment of unresectable cancer, achieving tumor ablation as well as side-effect reduction.

## Supplementary Material

Supplementary materials and figures.
