# Supplementary material for: A platelet-mimicking theranostic platform for cancer interstitial brachytherapy
Source: Theranostics. 2021 Jun 4;11(15):7589–99. doi: 10.7150/thno.61259 (PMC8210607; doi:10.7150/thno.61259)
Supplement: Supplementary file 1 — Supplementary materials and figures. [file thnov11p7589s1.pdf]

Supporting Information for

A platelet-mimicking theranostic platform for cancer interstitial brachytherapy

Meng Lyu[†], Mingzhu Chen[†], Lujie Liu[‡], Daoming Zhu[†,§], Xianjia Wu[‡], Yang Li[§,*], Lang Rao[‡,*] and Zhirong Bao[†,*]

[†] Department of Radiation and Medical Oncology, Hubei Key Laboratory of Tumor Biological Behaviors, Hubei Cancer Clinical Study Center, Zhongnan Hospital of Wuhan University, Wuhan 430071, China.

[‡] Institute of Biomedical Health Technology and Engineering, Shenzhen Bay Laboratory, Shenzhen 518132, China.

[§] Department of Gastrointestinal Surgery, Second Clinical Medical College of Jinan University, Shenzhen People's Hospital, Shenzhen 518020, China

* Corresponding e-mail: 1028168734@qq.com (Y.L.); lrao@szbl.ac.cn (L.R.); bao_zhirong@whu.edu.cn (Z.B.).

Experimental Section

Characterization

The microstructure images and element mapping were obtained by high-resolution transmission electron microscope (HRTEM; Tecnai G2 F20 STWIN, FEI, USA). XPS (ESCA-LAB250Xi, Thermo Fisher Ltd., USA) was used to analyze the surface chemical elements. The absorption spectra of samples were conducted on CARY5000 UV–visible–NIR spectrophotometer (Varian Ltd., USA). The zeta potential and zeta-diameter measurements were recorded on a dynamic light scatter (DLS, Nano-Zen 3600, Malvern Instruments, UK). CLSM images were captured using an IX 73 (Olympus, Japan).

Measurements of oxygen release

To detect the catalytic ability of nanomaterials in vitro, 1 mL of the nanomaterial aqueous solution including Au (0.03 mg/mL), CANS (0.03 mg/mL) and PLT/CANS (0.03 mg/mL) were incubated with 10 mL of H_2O_2 (30 mM) separately. The oxygen content was monitored real-time using a dissolved oxygen meter (HI9146, HANNA instruments, Korea). Photoacoustic imaging was conducted to measure the oxygenation ability in vivo on a Vevo®LAZR system (Fujifilm, Visualsonics Inc. Canada). The subcutaneous tumor bearing mice were intravenously (i.v.) administrated with PLT/CANS (10 mg/kg). PA images was recorded before injection and at various time post injection. Furthermore, for hypoxia detection in tumor tissue, orthotopic colon tumor mice were administrated with RBC/CANS or PLT/CANS via tail vein. The mice sacrificed 24 h post-injection, and the tumors were harvest, fixed in 4 % paraformaldehyde, followed by immunohistochemical staining of HIF-1α according to well-established protocols.

Cellular uptake

To assess the cellular uptake of nanomaterials, the CT 26 and RAW 264.7 cells were seeded in 6-well plates at the density of 1×10^5 cells per well. Then, nanomaterials were added into the medium and then washed with PBS. After incubation of 4 h, 1.5 ml of aqua regia was added in each well. Next, the samples were harvest and an

inductively coupled plasma-atomic emission spectrometer (ICP-AES) was used to determine the Au content in each sample.

Colony formation assay

CT-26 cells were seeded in 6-well plates at a density of 500 cells/well. After being cultured with nanomaterials at various concentration for 6 h, cells were subjected to irradiation of X-rays. Then the cells were washed for several times using saline. After cells were cultured in fresh medium for 15 days, crystal purple was utilized for dyeing the cells.

Western blot

Western-blot analysis was performed to analyze the level of protein from cells in each groups. The cells were lysed in RIPA buffer containing protease inhibitors and phosphatase inhibitor (Sigma-Aldrich). Bradford protein assay (Bio-Rad Ltd., Germany) was performed to measure the protein concentration. The total protein was isolated and subjected to 10% SDS-PAGE (Bio-Rad Ltd., Germany), and transferred to a PVDF membrane (Millipore Ltd., USA). Afterward, TBST(20mMTris,150mMNaCl) with 5% milk was used to block non-specific binding sites. The membranes were then incubated with the primary antibodies (Bethyl Laboratories,Inc.) in 4 °C overnight. After washing and further incubation with appropriate secondary antibodies (Jackson Immuno Research Laboratories) for 1 hour at room temperature. The signal was detected using an enhanced chemiluminescence system (ECL; Amersham).

Specific-site targeting and accumulation of PLT/CANS

The orthotopic colon tumor mice were i.v. injected with the Cy-5-labeled RBC/CANS (10 mg/kg) and PLT/CANS (10 mg/kg) via tail vein. Then the mice were sacrificed after 24 h. Main organs and tumors were harvest and imaged by Maestro Automated in vivo imaging system (Caliper, USA).

For PA imaging, the mice with subcutaneous tumor were i.v. injected with RBC/CANS (10 mg/kg) and PLT/CANS (10 mg/kg) respectively. Before and 0 h, 24 h after the injection, PA images were obtained.

Pharmacokinetics study

Balb/c mice received i.v. injection of CANS, RBC/CANS and PLT/CANS at same dosage of 10 mg/kg. At 0.5 h, 1 h, 4 h, 8 h, 12 h and 24 h post injection, 15 μL blood samples were collected and lysed with aqua regia for Au quantification by ICP-AES. 60 days post injection, mice were sacrificed. Blood samples and main organs were harvest for blood and histological analysis.

Biodistribution

14 days post the establishment of orthotopic colon tumor, mice were separated into two groups and i.v. injected with Cy5-labeled RBC/CANS (10 mg/kg) and Cy5-labeled PLT/CANS (10 mg/kg) respectively. For biodistribution study, mice were anaesthetized and sacrificed. Main organs as well as tumors were imaged using fluorescence imaging system, followed by collected, weighed, lysed with aqua regia and Au content was measured using ICP-AES.

Supplementary Figures

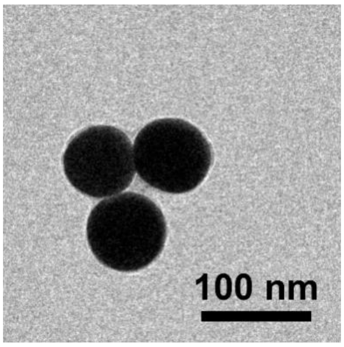

Figure S1. TEM images of gold nanoparticles.

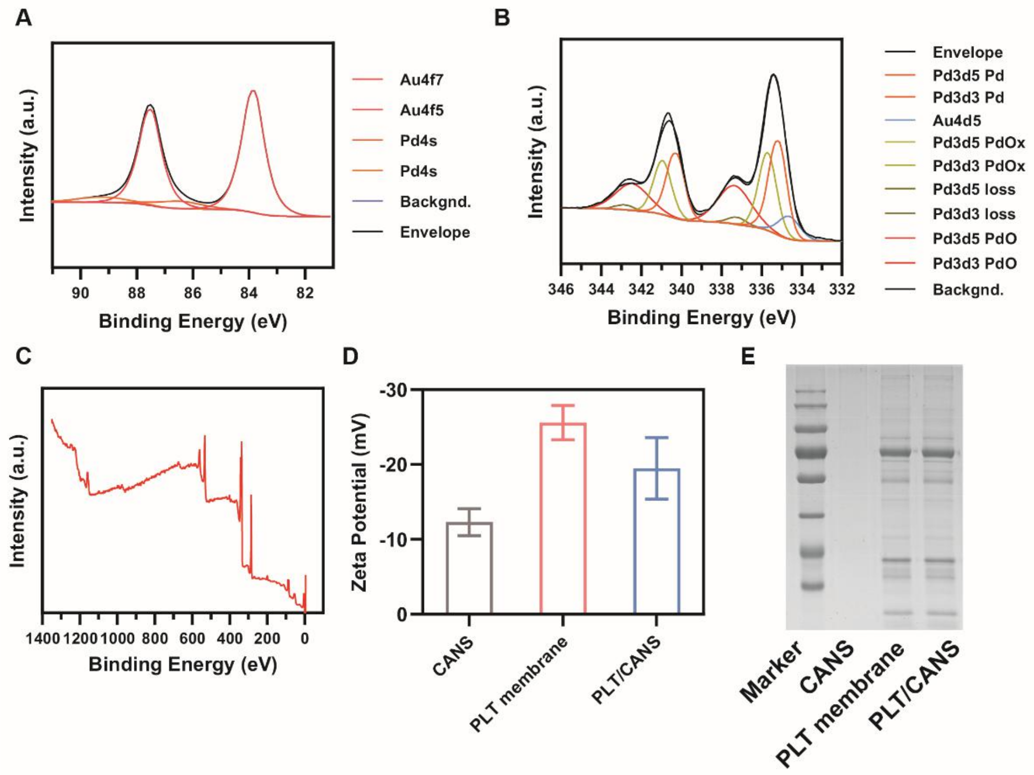

Figure S2. Characterization of CANS and PLT/CANS. High resolution XPS spectra of CANS of A) Au orbit and B) Pd orbit. C) XPS spectrum of CANS. D) Zeta potential of CANS, PLT membrane and PLT/CANS. E). SDS-PAGE of CANS, PLT membrane and PLT/CANS.

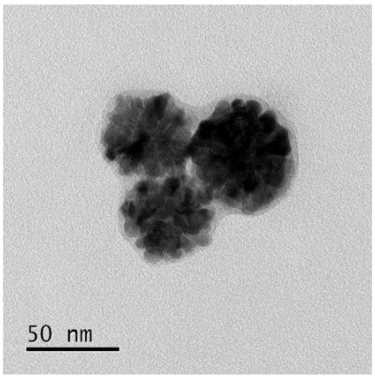

Figure S3. TEM image of RBC/CANS.

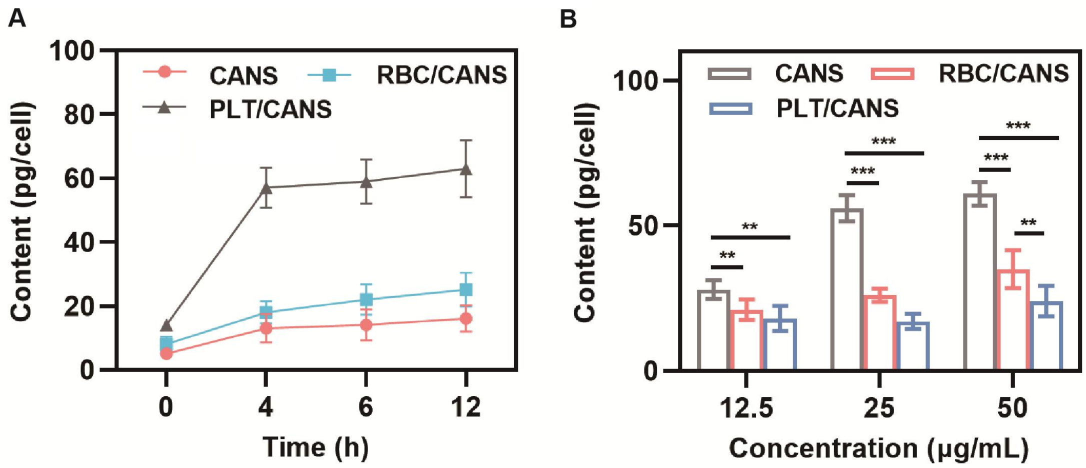

Figure S4. Au levels in A) CT 26 cells after various treatment of different concentrations with various incubation durations at a concentration of 50 μg/mL and B) RAW 264.7 cells after various treatment of different concentrations. * $P < 0.1$; ** $P < 0.01$; *** $P < 0.001$.

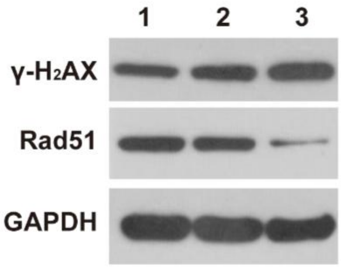

Figure S5. Western blot analysis. 1: RT; 2: RBC/CANS+RT; 3: PLT/CANS+RT.

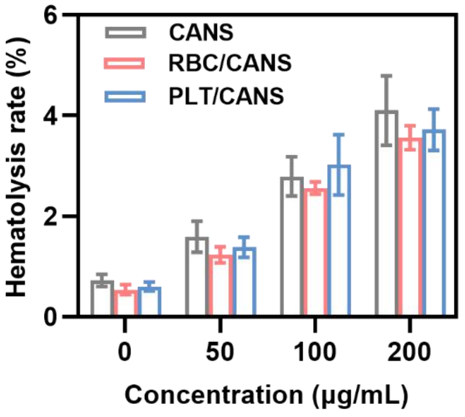

Figure S6. Hemolysis tests of CANS, RBC/CANS and PLT/CANS.

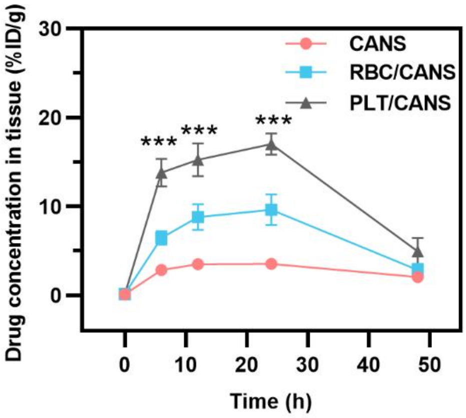

Figure S7. Tumor accumulation of Au at various time post injection of CANS, RBC/CANS and PLT/CANS. * P < 0.1; ** P < 0.01; *** P < 0.001.

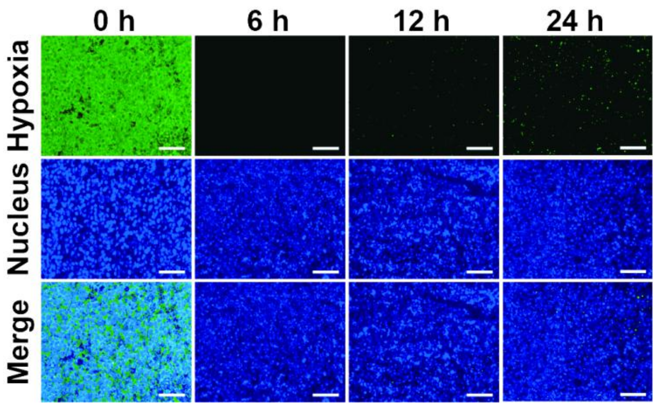

Figure S8. HIF-1α stained images of tumors at various time post injection of PLT/CANS (Scale bar: 50 μm).
